# Research trends and hotspots of ketamine from 2014 to 2023: a bibliometric analysis

**DOI:** 10.3389/fnins.2024.1407301

**Published:** 2024-06-14

**Authors:** Yida Wang, Sile Chang, Dongxu Chen

**Affiliations:** ^1^Key Laboratory of Bio-Resource and Eco-Environment of Ministry of Education, College of Life Science, Sichuan University, Chengdu, China; ^2^Department of Anesthesiology, West China Second Hospital, Sichuan University, Chengdu, China; ^3^Key Laboratory of Birth Defects and Related Diseases of Women and Children (Sichuan University), Ministry of Education, Chengdu, China

**Keywords:** ketamine, bibliometric analysis, depression, anesthesia, analgesic

## Abstract

**Background:**

Despite this growing interest, there remains a lack of comprehensive and systematic bibliometric analyses of ketamine research. This study aimed to summarize the progress in ketamine research through bibliometric analysis, providing insights into the development and direction of the field.

**Methods:**

Publications related to ketamine were retrieved from the Web of Science Core Collection (WoSCC) database on February 15, 2024. In conducting a comprehensive bibliometric analysis, a variety of bibliographic elements were meticulously collected to map the landscape of research within a specific field.

**Results:**

Between January 1, 2014, and December 31, 2023, a total of 10,328 articles on ketamine research were published across 1,752 academic journals by 45,891 authors from 8,914 institutions in 128 countries. The publication volume has shown a steady increase over this period. The United States of America (USA) and the People’s Republic of China lead in both publication and citation counts. The National Institute of Mental Health (NIMH) and Yale University emerge as the most active institutions in this research domain. Carlos Zarate of the NIH National Institute of Mental Health was noted for the highest number of significant publications and received the most co-citations. The analysis revealed key research themes including mechanism of action, adverse events, psychiatric applications, and perioperative implications.

**Conclusion:**

This study provided comprehensive bibliometric and knowledge mapping analysis of the global ketamine research landscape, offering valuable insights into the trends, key contributors, and thematic focus areas within the field. By delineating the evolution of ketamine research, this study aims to guide future scholarly endeavors and enhance our understanding of ketamine’s therapeutic potential.

## 1 Introduction

Originally developed as an anesthetic, ketamine has gained prominence for its rapid and potent antidepressant effects in recent years. The exact mechanism underlying these antidepressant effects remains complex and incompletely understood (Gould et al., 2019). It is hypothesized that ketamine’s rapid antidepressant action is primarily mediated through its antagonism of N-methyl-D-aspartate (NMDA) receptors, leading to enhanced synaptic plasticity and synaptogenesis in critical brain regions involved in mood regulation, such as the prefrontal cortex and hippocampus (Zanos et al., 2023). Additionally, ketamine is believed to augment the function of alpha-amino-3-hydroxy-5-methyl-4-isoxazolepropionic acid (AMPA) receptors, promoting synaptic potentiation (Zaytseva et al., 2023). Other proposed mechanisms include the regulation of the mammalian target of rapamycin (mTOR) signaling pathway, increased expression of brain-derived neurotrophic factor (BDNF), and the reduction of inflammation and oxidative stress, further contributing to its antidepressant effects (Li et al., 2010; Autry et al., 2011; Gao et al., 2022). Ketamine stands out for its ability to quickly alleviate symptoms in individuals experiencing severe, treatment-resistant episodes of depression. Its distinct mode of action not only promises to deepen our understanding of depression’s pathophysiology but also serves as a foundation for creating innovative antidepressant medications that are equally effective but have fewer side effects. Beyond its use in depression, ketamine has shown efficacy in alleviating symptoms of other mood disorders, such as bipolar depression and post-traumatic stress disorder (PTSD) (Yavi et al., 2022). Early evidence also indicates a potential reduction in suicidal thoughts among those treated, positioning ketamine infusion therapy as a promising strategy for addressing acute suicidal ideation. This multifaceted potential emphasizes ketamine’s significance in the landscape of psychiatric treatment, highlighting its role as a catalyst for developing broader therapeutic options (Abbar et al., 2022).

While ketamine’s potential as a therapeutic agent is significant, its use is accompanied by various adverse effects that necessitate cautious clinical application (Rolfzen et al., 2024). During ketamine infusion, common acute side effects such as dissociation, hallucinations, dizziness, and transient increases in blood pressure and heart rate may occur (Weiner et al., 2000; Timm et al., 2008; Kalsi et al., 2011; Guglielminotti et al., 2019; Abdoullateef et al., 2024). Prolonged or frequent use can lead to more severe consequences, including cognitive decline, urinary and gastrointestinal issues, as well as the risk of abuse and dependency (Weiner et al., 2000; Morgan and Curran, 2006; Cvrček, 2008; Kalsi et al., 2011; Niesters et al., 2014). Furthermore, the long-term safety profile of ketamine, especially concerning neurodevelopment and cognitive function, remains under-researched and demands further investigation.

The clinical deployment of ketamine therapy faces numerous hurdles. The lack of standardized guidelines for dosage, administration frequency, and treatment duration contributes to inconsistent clinical outcomes and elevates potential risks (Niesters et al., 2014). Concerns regarding ketamine’s potential for abuse and diversion underscore the necessity for strict regulatory frameworks and vigilant monitoring practices. Although a number of academic reviews on ketamine have been published in recent years, providing relatively comprehensive descriptions, these reviews have focused on overall trends in published data in the literature based only on bibliometric analyses, and there has been a lack of discussion of the keyword drug itself behind these data, and in particular a lack of attention given to ketamine in terms of the clinical use of the drug and the safety adverse events (He et al., 2022; Miao et al., 2022). To enhance the understanding and facilitate the research progression in the realm of ketamine therapy, this study undertook a comprehensive analysis of the existing literature on ketamine studies. The goal was to pinpoint emerging trends and hotspots within ketamine research, thereby propelling forward the exploration and knowledge in this critical area of psychiatric treatment.

## 2 Methods

### 2.1 Search strategy and data collection

We conducted a systematic review of literature on ketamine within the Web of Science Core Collection (WOSCC) for the years 2014 to 2023. Utilizing the advanced search capabilities of WOSCC, we aimed to ensure the representativeness and generalizability of our findings. Our search focused on the terms’ “ketamine,” specifically targeting research articles and review papers while excluding other article types. This search, completed on February 15, 2024, resulted in a comprehensive dataset exported in plain text format for further analysis. We did not use any exclusion criteria.

### 2.2 Data analysis

The dataset included diverse parameters such as the geographical distribution of contributions, affiliating institutions, publishing journals, individual authors, abstracts, and citation frequencies. Additionally, we assessed the 2022 Journal Citation Report (JCR) category for each included journal. Using objective and quantitative indicators extracted from the Web of Science database, such as citation counts and the H-index, we evaluated the scientific output and academic impact of various researchers and groups.

For detailed analysis, we employed VOSviewer 1.6.20 for conducting co-authorship analyses at the levels of country/region, institution, and individual authors, along with an analysis of keyword contributions. The networks visualized by VOSviewer featured nodes representing the volume of publications, where larger nodes indicated higher publication output. The links between nodes illustrated the relationships among the various parameters, with the thickness of the links indicating the strength of these relationships.

Furthermore, we conducted a journal co-authorship analysis using CiteSpace 6.3.R1, spanning the research period from 2014 to 2023 and applying a year-wise time-slicing method. This analysis considered all available source terms and selected node types based on specific criteria related to the g-index. The resulting graphical representation detailed observations including countries, institutions, authors, co-cited references, and keywords. In cases of multi-country, −institution, and -author collaborations, each was uniquely represented within the analysis, providing a comprehensive view of the research landscape and collaborative efforts in the field of ketamine research.

## 3 Results

### 3.1 Trends in publications

**Figure 1:**
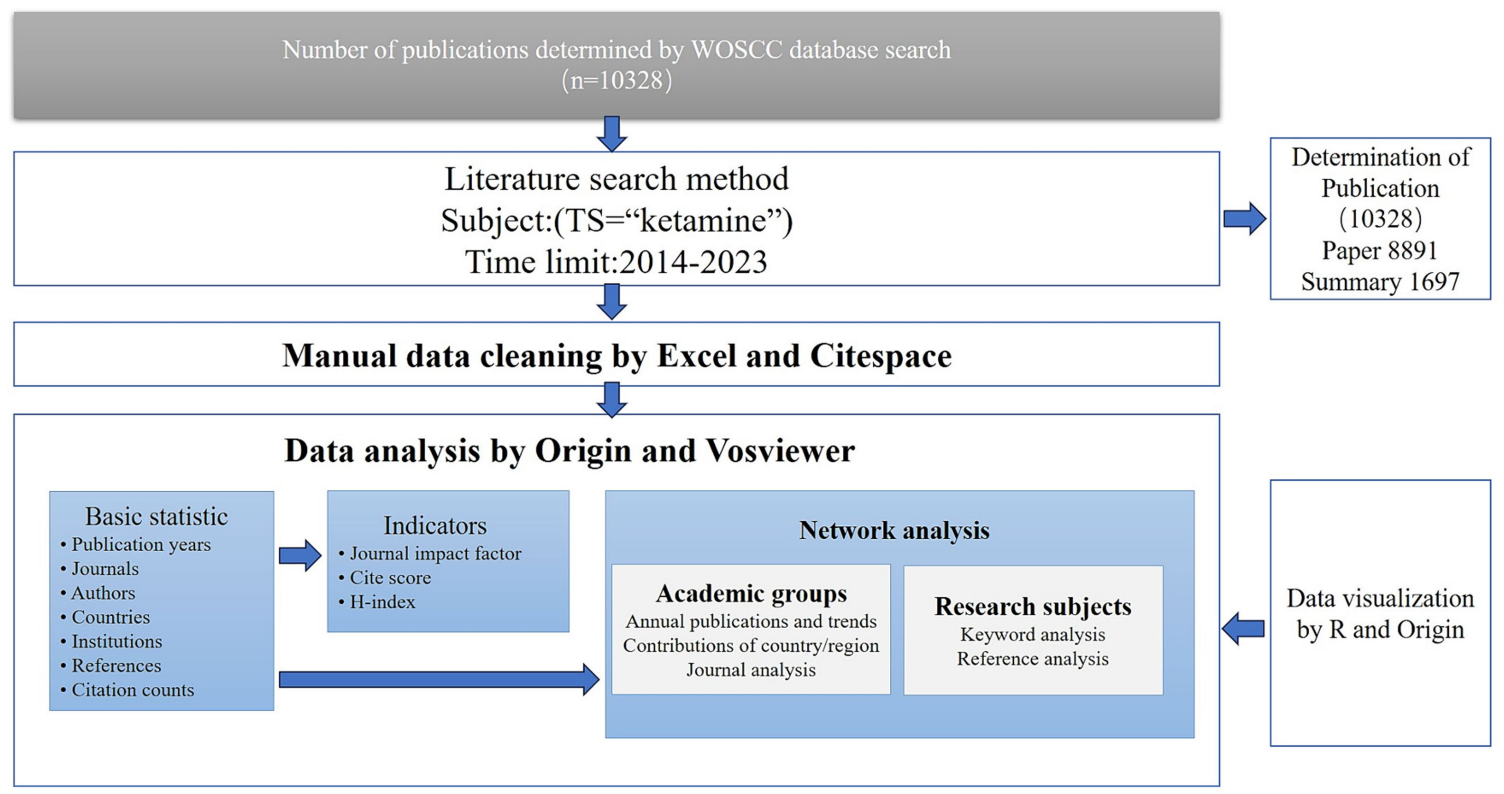
Flowchart of the screening process of ketamine research.

**Figure 2:**
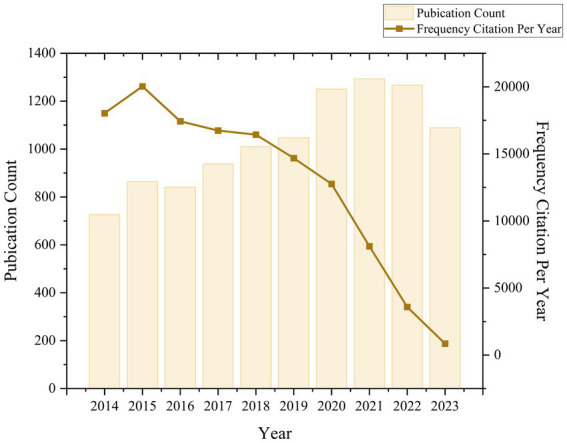
Global trend and continuous growth rate of annual publications related to ketamine from 2014 to 2023.

Our search in the WOSCC database identified 10,328 publications eligible for bibliometric analysis spanning the last decade (2014–2023) (Figure 1). Analysis of the citation report from the WOSCC database revealed a total of 130,275 citations, with 128,664 citations when excluding self-citations, averaging 12.61 citations per publication (Figure 2). Over this ten-year period, research on ketamine has demonstrated a general upward trajectory in publication volume. Notably, the years 2019–2020 experienced the highest growth rate in publications over the last decade, at 19.34%. The period from 2020 to 2022, with an annual publication count ranging from 1,250 to 1,293 articles, marked the decade’s peak in publication volume, with the highest number reaching 1,293 in 2021. Additionally, the interval between 2020 and 2021 (from 17,944 to 25,489 annual citations) represented the most rapid citation growth over 15 years, with an increase of 1,747 citations. These findings underscore the escalating interest and investment in research related to ketamine, indicating a growing recognition of the field’s significance.

### 3.2 Country/region contribution analysis

**Figure 3:**
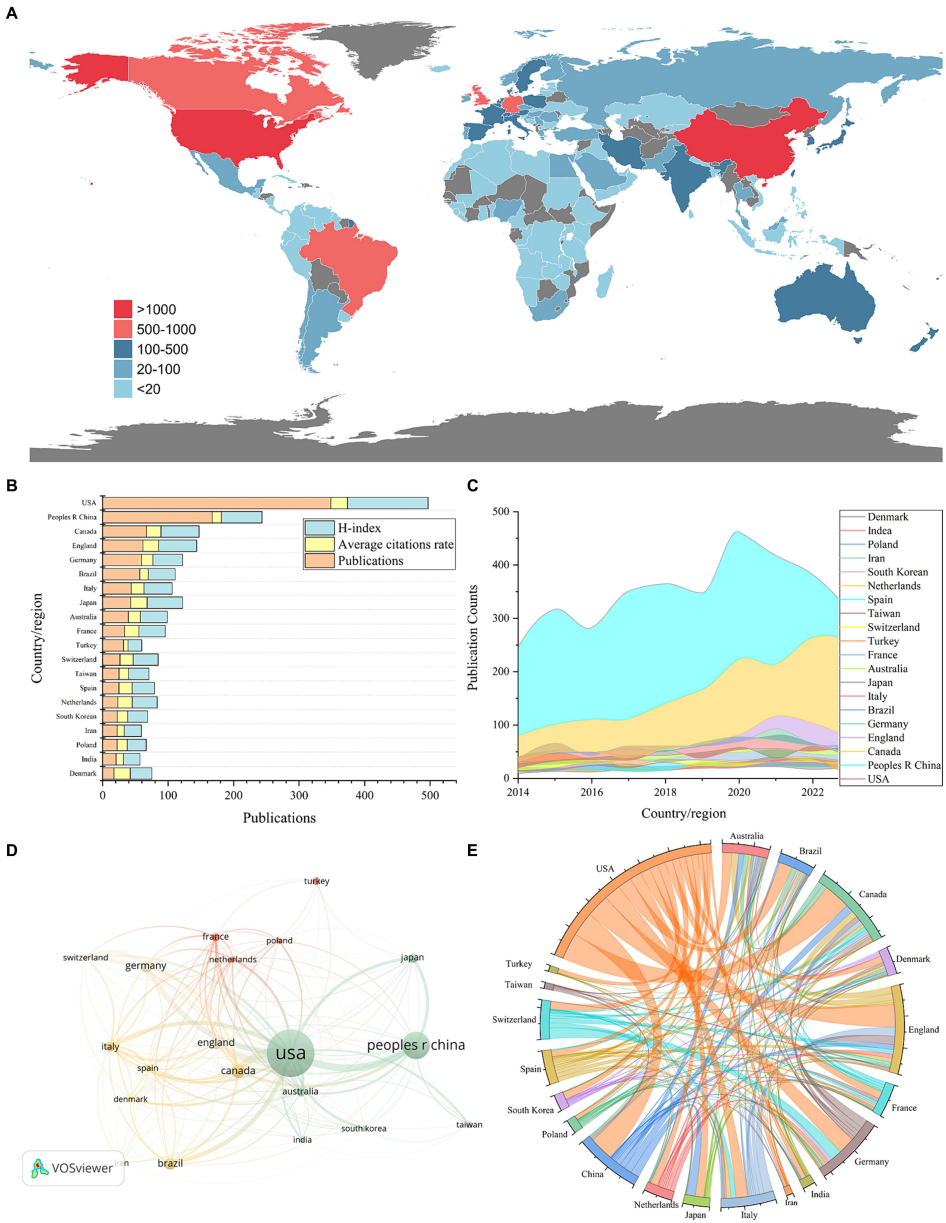
National and regional contributions to the field of ketamine research are depicted in following aspects: **(A)** Global map illustrating the distribution of research on neural circuits, with different countries represented by distinct colors based on their publication counts. **(B)** The top 20 countries’ publication output in this field (displayed as total number divided by ten), along with average citations per paper and the H-index. **(C)** An analysis of international collaboration and participation among countries/regions in ketamine studies, where line thickness indicates cooperation frequency. **(D)** Noticeable upward trend in the number of papers published by the top 20 countries/regions in ketamine research from 2014 to 2023. **(E)** The string diagram reflects the cooperation between countries or regions, and the width of the lines reflects the closeness of cooperation between two countries or regions.

Through the application of VOSviewer for analyzing country contributions in ketamine research and employing the R package for data visualization, we mapped the global distribution of publications (Figure 3A). Setting a minimum publication threshold of 200 articles for country-level analysis, 20 countries met this criterion. The United States led in publication volume with 3,485 articles, followed by China with 1,672, and Canada with 667 (Table 1 and Figures 3B,C). Our analysis found that 129 countries have engaged in collaborative efforts in this field (Figures 3D,E).

**Table 1:** Top 20 countries/regions that have published research publications on ketamine research.

Country/region	Publications	Citations	Average citations rate	H-index
USA	3,485	88,670	25.44	123
Peoples R China	1,673	23,550	14.08	62
Canada	667	14,990	22.47	58
England	616	14,718	23.89	58
Germany	593	10,412	17.56	45
Brazil	567	7,334	12.93	41
Italy	436	8,530	19.56	43
Japan	428	10,786	25.2	54
Australia	392	7,212	18.4	41
France	335	7,351	21.94	40
Turkey	318	2,206	6.94	21
Switzerland	265	5,344	20.17	38
Taiwan	250	3,663	14.65	31
Spain	249	5,019	20.16	34
Netherlands	231	5,113	22.13	38
South Korean	223	3,558	15.96	30
Iran	222	2,405	10.83	26
Poland	220	3,415	15.52	29
India	204	2,354	11.54	25
Denmark	172	4,311	25.06	33

When evaluating the average number of citations per article, the United States also ranked highest with an average of 25.44 citations per article, closely followed by Japan with 25.2 and Denmark with 25.06 (Table 1 and Figures 3B,C). Additionally, in terms of the H-index, which measures both the productivity and citation impact of the publications, the United States achieved the highest score of 123. China, Canada, and England followed with H-index scores of 62, 58, and 58, respectively. This comprehensive analysis highlights the significant contributions of these countries to the body of knowledge in ketamine research and underscores the global interest and collaborative nature of this scientific inquiry.

### 3.3 Institutional distribution analysis

**Figure 4:**
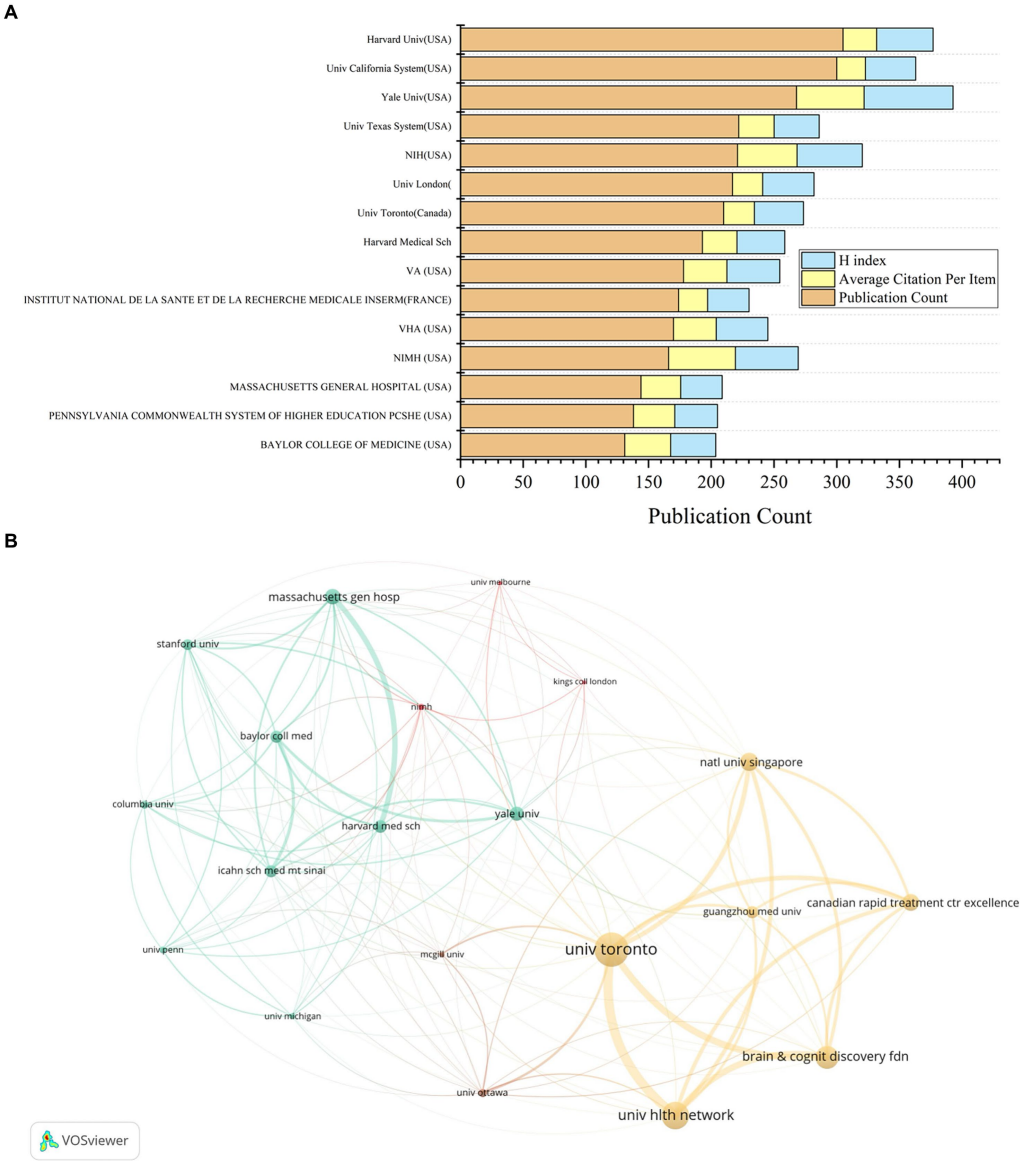
Contributions by institutions in the field of ketamine research. **(A)** The total number of papers, average citations and H-index of the top 15 publications in the field. **(B)** A co-analysis chart of the top 20 institutions in the ketamine research field. Each node represents an institution, and the node size represents the number of papers published by institution. The connection between nodes indicates cooperation, and the width of the connecting lines indicates the numbers of cooperation.

The analysis of the top 20 institutions contributing to ketamine research reveals their geographical distribution across the United States, Canada, the United Kingdom, and France. Harvard University leads with 295 publications, closely followed by the University of California with 288, and Yale University with 259 (Table 2 and Figure 4A). Notably, From the citation analysis function of WOS, it can be seen that Harvard University has shown a consistent annual increase in publications from 2018 to 2022, with a peak in 2022. Similarly, significant publication activity was observed at the University of California and Yale University in 2020.

**Table 2:** The top 15 institutions with the most publications in the field of ketamine research.

Institute	Publication Count	Average Citation Per Item	H index
Yale Univ (USA)	259	53.56	70
NIH (USA)	215	48.51	52
NIMH (USA)	160	54.89	49
Harvard Univ (USA)	295	27.72	45
Univ London (England)	204	24.73	41
Univ California System (USA)	288	23.77	40
VA (USA)	168	31.93	40
Univ Toronto (Canada)	208	25.27	39
Harvard Medical Sch (USA)	189	28.42	38
VHA (USA)	160	31.18	37
Univ Texas System (USA)	217	28.83	36
Baylor College of Medicine (USA)	129	36.72	36
Pennsylvania State System of Higher Education (USA)	134	34.61	35
Institut national de la santé et de la recherche médicale (FRANCE)	173	23.61	33
Massachusetts General Hospital (USA)	140	32.5	32

When examining citations per article, the National Institute of Mental Health (NIMH) ranks highest with an average of 54.89 citations per article, followed by Yale University with 53.56, and the National Institutes of Health (NIH) USA with 48.51. In terms of the H-index, a metric that assesses both the productivity and impact of the published work, Yale University leads with a score of 70, with NIH at 52 and NIMH at 49 (Table 2 and Figure 4A).

Mapping the top 20 regions by publication volume highlights strong intra-institutional collaborations within the United States (Figure 4B). Meanwhile, the University of Toronto stands out for its extensive international collaborations, notably with the National University of Singapore, Guangzhou Medical University, and the Hltm Network University. This pattern underscores the global nature of ketamine research, highlighting the significance of both domestic and international collaborations in advancing the field.

### 3.4 Analysis of journal distribution

From 2014 to 2023, a total of 128 journals contributed research papers to this area. An overview of the top 10 journals, as shown in Table 3, reveals that the majority (70%, 7 out of 10) are categorized within the Q2 quartile, indicating a strong presence of reputable, though not the highest-tier, journals in disseminating ketamine research. Veterinary Anaesthesia and Analgesia leads with the highest number of publications, totaling 166 papers. Notably, Frontiers in Psychiatry emerged as the most prolific journal from 2021 to 2023, publishing a total of 93 articles in this period. This suggests a growing interest and focus on the psychiatric implications of ketamine research in recent years. Table 4 lists the top 10 most cited references in this domain, underscores the influence of high-impact publications, with Nature contributing 20% (2 out of 10) of these seminal works. This highlights the significant scientific interest and the critical reception of ketamine research within the broader scientific community, particularly in high-profile journals known for their rigorous selection criteria and wide-reaching impact.

**Table 3:** The influence index of the top 10 journals with the largest number of articles related to ketamine.

Journal	Publication count	Impact factor(2022)	Journal citation reports	Total citations	Average citation per item
Veterinary Anaesthesia and Analgesia	166	1.7	Q2	967	7.87
Journal of Affective Disorders	149	6.6	Q1	3,546	23.8
Psychopharmacology	140	3.4	Q2	3,192	22.8
Neuropharmacology	136	4.7	Q2	3,455	25.4
Frontiers in Psychiatry	134	4.7	Q2	1,682	12.55
Plos One	129	3.7	Q2	2070	16.05
Neuropsychopharmacology	109	6.7	Q1	4,072	37.36
Scientific Reports	107	4.6	Q2	1940	18.13
Behavioural Brain Research	93	2.7	Q2	1,652	17.76
International Journal of Neuropsychopharmacology	91	4.8	Q1	3,303	36.3

**Table 4:** The top 10 most cited references in this domain in the field of ketamine research.

Title	Corresponding author	Journal	IF	Publication year	Total citations	Research direction
NMDAR inhibition-independent antidepressant actions of ketamine metabolites	Zanos, Panos	NATURE	64.8	2016	1,004	Multidisciplinary Sciences
Prevention and Management of Chemotherapy-Induced Peripheral Neuropathy in Survivors of Adult Cancers: American Society of Clinical Oncology Clinical Practice Guideline	Hershman, Dawn L.	JOURNAL OF CLINICAL ONCOLOGY	45.4	2014	813	Oncology
BDNF - a key transducer of antidepressant effects	Bjorkholm, Carl	NEUROPHARMACOLOGY	4.7	2016	607	NeurosciencesPharmacology & Pharmacy
Ketamine and Ketamine Metabolite Pharmacology: Insights into Therapeutic Mechanisms	Zanos, Panos	PHARMACOLOGICAL REVIEWS	21.1	2018	581	Pharmacology & Pharmacy
Ketamine blocks bursting in the lateral habenula to rapidly relieve depression	Yang, Yan	NATURE	64.8	2018	564	Multidisciplinary Sciences
Mechanisms of ketamine action as an antidepressant	Zanos, P.	MOLECULAR PSYCHIATRY	11	2018	527	Biochemistry & Molecular BiologyNeurosciencesPsychiatry
Effects of Psilocybin-Assisted Therapy on Major Depressive Disorder A Randomized Clinical Trial	Davis, Alan K.	JAMA PSYCHIATRY	25.8	2021	511	Psychiatry
Clinical Electroencephalography for Anesthesiologists Part I: Background and Basic Signatures	Purdon, Patrick L.	ANESTHESIOLOGY	8.8	2015	442	Anesthesiology
Psychedelics Promote Structural and Functional Neural Plasticity	Ly, Calvin	CELL REPORTS	8.8	2018	432	Cell Biology
Ketamine and Other NMDA Antagonists: Early Clinical Trials and Possible Mechanisms in Depression	Newport, DJ	AMERICAN JOURNAL OF PSYCHIATRY	17.7	2015	405	Psychiatry

### 3.5 Author distribution analysis

**Figure 5:**
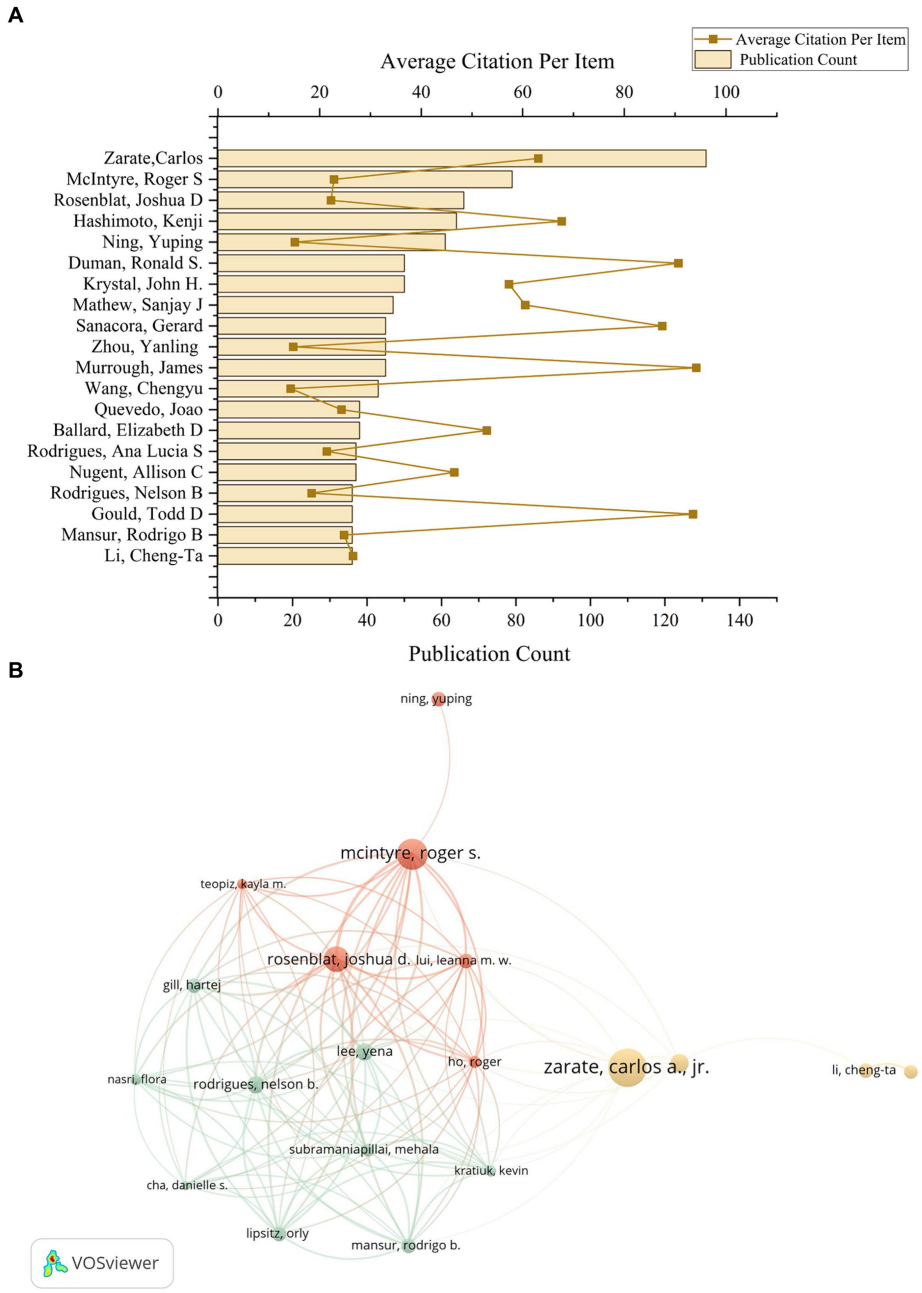
The author’s contributions to the ketamine research field. **(A)** The total number of papers by the top 20 authors who contributed the most in the field and the average number of citations per paper. **(B)** According to VOSviewer, a co-analysis diagram between these 20 authors. Each node represents an author, and the node size represents the number of published papers. The connections between nodes indicate that there is collaboration between authors, and the width of the lines indicates the number of collaborations.

In the United States, Carlos Zarate of the NIH stands out as a leading figure in ketamine research, contributing to a total of 131 publications and receiving the highest citation count of 8,261 (Figure 5A). This underscores Zarate’s substantial influence and the critical reception of his work in advancing our understanding of ketamine’s therapeutic potentials. Meanwhile, Kenji Hashimoto from Chiba University in Japan emerges as the most prolific author in ketamine research within Japan, with a publication count of 64 articles and 4,330 citations. These figures not only highlight the significant contributions of these researchers to the ketamine field but also illustrate the global recognition of their work, marking them as key influencers in the ongoing exploration of ketamine’s clinical applications. Based on author and co-author information, we constructed a collaboration network, which allows visualization of the degree of scientific collaboration concentration between authors by using co-authorship frequency (Figure 5B).

### 3.6 Keyword co-occurrence cluster analysis

**Figure 6:**
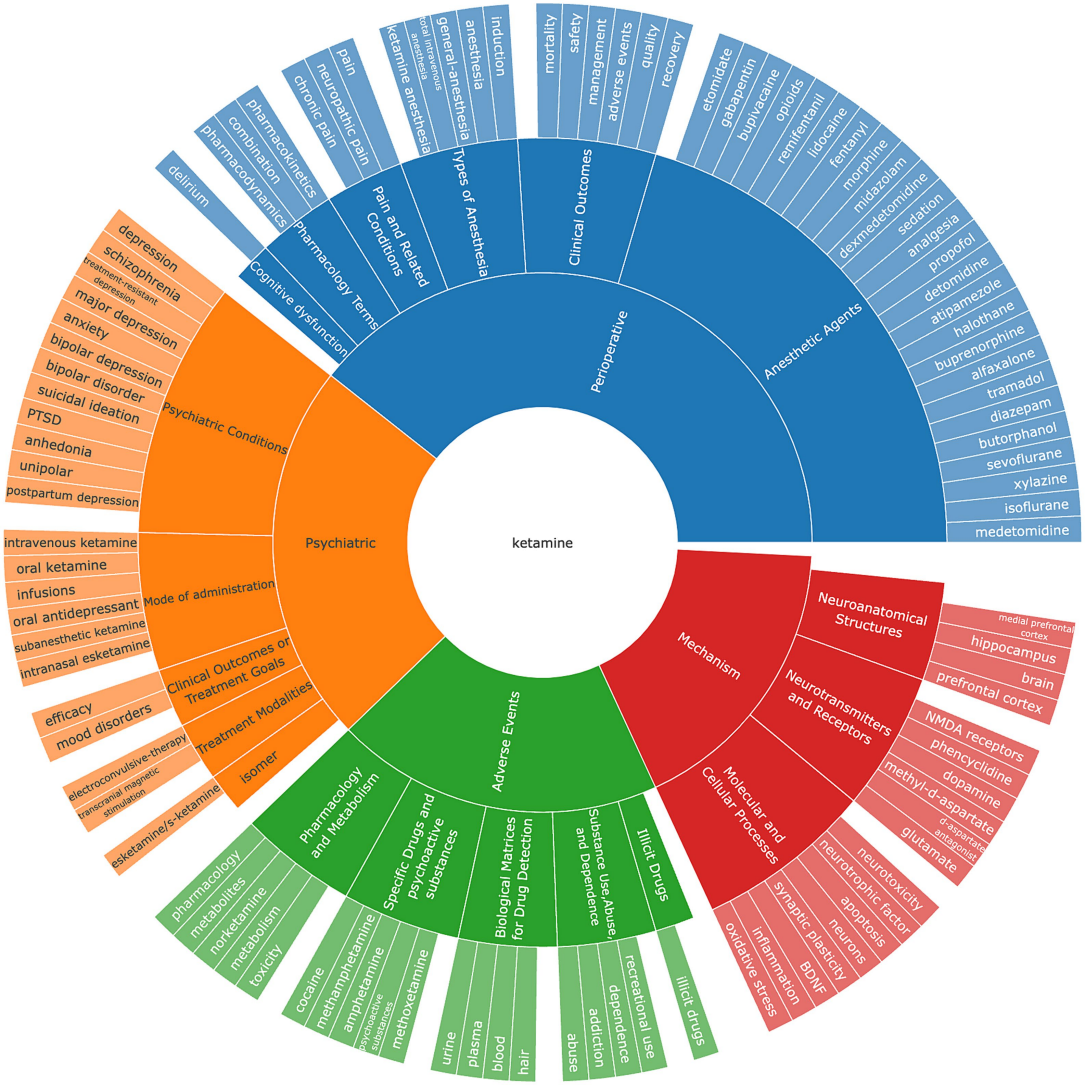
The keywords classification on ketamine research field. Torus of keyword classification clusters and groups high-frequency keywords according to different fields.

After meticulously processing the 10,328 eligible documents, the VOSviewer tool was utilized to filter out keywords with less than five occurrences. These keywords were clustered, analyzed, and then manually sorted into categories, culminating in the creation of a keyword ring illustrated in Figure 6. The categorization process divided the keywords into four primary groups: mechanism, adverse events, psychiatric applications, and perioperative implications.

Within the “mechanism” category, keywords predominantly encompass Neuroanatomical Structures, Neurotransmitters and Receptors, and Molecular and Cellular Processes. This highlights the focus on understanding the intricate biological and chemical foundations of ketamine’s action. The “adverse events” category primarily deals with issues related to drug abuse, psychotropic substances, and dependency, alongside pharmacological mechanisms. This reflects the concern over the potential misuse of ketamine and the need for understanding its pharmacological profile to mitigate such risks. On the “psychiatric” front, the emphasis is on conditions treated with ketamine, including anxiety, suicidal ideation, and PTSD. This underscores the therapeutic potential of ketamine in addressing severe psychiatric conditions. The “perioperative” category includes keywords related to ketamine’s administration methods (e.g., injections, oral, or intranasal) and its use in surgical contexts. Here, ketamine’s role as an anesthetic and its comparison with other anesthetics like morphine and fentanyl are of particular interest. The clinical concerns revolve around mortality, safety, indications, and the adverse effects of delirium, highlighting the importance of cognitive function post-operation. Additionally, the research explores ketamine’s differential impact and side effects across various anesthesia methods, targeting specific areas and patient groups. The analgesic properties of ketamine, its effectiveness in pain management, and related research areas also receive significant attention. From a pharmacological perspective, the interactions between ketamine and other drugs, along with its pharmacodynamics and pharmacokinetics, are current focal points. This comprehensive keyword analysis reveals the multifaceted research landscape surrounding ketamine, from its basic mechanisms of action to its clinical and pharmacological implications, illustrating the broad scope of investigation into this versatile drug.

**Figure 7:**
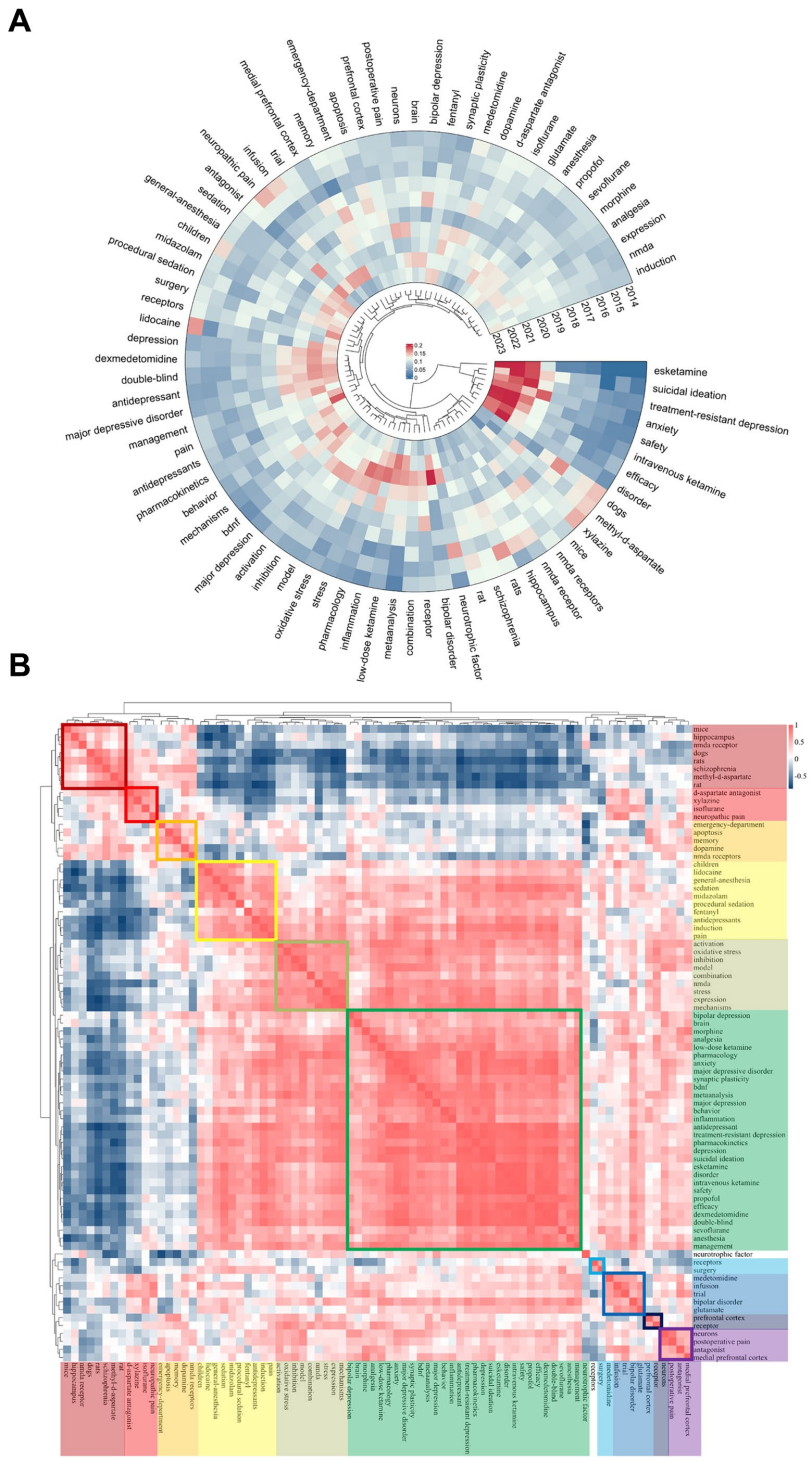
Ketamine keyword heat map analysis. **(A)** Heat maps generated for ketamine studies, measuring the popularity of a keyword by its citation ratio within that year. **(B)** Ketamine-related heat map categorizes the most popular keywords from the same period using different colors.

Figure 7A presents the top 80 most relevant keywords for ketamine research from 2014 to 2023, highlighting the annual distribution of these keywords by measuring the ratio of citations per keyword to the total citations within the same year. In the most recent three-year span, keywords such as esketamine, suicidal ideation, treatment-resistant depression, anxiety, safety, intravenous ketamine, efficiency, and disorder emerged with the highest annual popularity, signaling their ascent as the focal points of contemporary research within the ketamine discourse. This shift underscores a growing interest in specific aspects of ketamine research, notably its application in treating severe psychiatric conditions and ensuring the safety and efficacy of its use. Interestingly, while keywords like esketamine and anxiety have surged in prevalence, others related to memory, the emergency department, and apoptosis have seen a decline in relative prominence. Furthermore, topics like bipolar disorder, inflammation, and meta-analysis, particularly notable between 2020 and 2022, continue to hold significant relevance in the exploration of ketamine’s potential.

Figure 7B maps the popularity correlation among keywords, arranging them into ten distinct clusters based on their co-occurrence and thematic similarity during the same period, each marked by a unique color. These clusters range from neurobiological mechanisms (dark red cluster featuring mice, hippocampus, NMDA receptors) to clinical applications (green cluster with morphine, analgesia, pharmacology) and specific research methodologies (dark blue cluster with medetomidine, infusion, trial). This classification not only elucidates the diverse areas of interest within ketamine research but also illustrates how these focal areas are interlinked, reflecting the multifaceted nature of current investigations into ketamine’s therapeutic potentials and challenges.

## 4 Discussion

### 4.1 Main findings

Ketamine, known for its analgesic properties without the risk of narcotic dependence and its broad inhibitory effects on the central nervous system, has found extensive use in critical medical areas. These include its role in surgical anesthesia, the management of both acute and chronic pain, and its use as a rapidly acting antidepressant. This research endeavor analyzed 10,328 documents related to ketamine, extracted from the WOSCC database, covering publications from 2014 to 2023. The upward trajectory in publication volume during this period underscores a growing scholarly interest in ketamine, highlighting its significance across various medical disciplines. Our findings underscore the geographical variations in ketamine research, advocating for the necessity of collaborative efforts that span multiple countries and institutions. This collaborative approach is crucial for advancing our understanding of ketamine’s multifaceted roles. Furthermore, through an in-depth examination of co-citations and keyword trends, this study delineates the current research landscape, focusing on ketamine’s mechanisms of action, associated adverse events, its psychiatric applications, and its utility in perioperative settings. This ground-breaking analysis provides a comprehensive overview of ketamine’s clinical applications, charting the course of its scientific exploration over the last decade. The insights garnered from this study are invaluable, offering a roadmap for both researchers and clinicians to navigate the complexities of ketamine’s use, with the ultimate goal of enhancing clinical outcomes and fostering future scientific inquiries into this versatile pharmacological agent.

### 4.2 Research progress and evolving trend

The trajectory of ketamine research from 2014 to 2023 illustrates a dynamic and evolving field, marked by shifts in focus from basic pharmacological properties to sophisticated clinical applications and mechanisms of action.

#### 4.2.1 2014–2016: initial focus on anesthesia and pain management

During this period, research predominantly centered on ketamine’s anesthetic properties and its efficacy in pain relief, particularly in clinical settings. The combination effects of ketamine with other drugs like lidocaine and xylazine were explored, alongside its role in managing neuropathic pain symptoms, using rats and dogs as the primary model organisms.

#### 4.2.2 2017–2019: shift towards mechanistic understanding

Research emphasis transitioned to understanding ketamine’s mechanism of action, with a significant focus on its interaction with NMDA receptors and effects on the medial prefrontal cortex. This era saw a deep dive into ketamine’s potential for treating specific types of depression, notably bipolar depression, and its impact on the brain’s dopamine system, suggesting ketamine-induced structural changes that modulate emotion regulation.

#### 4.2.3 2020 onwards: clinical application and efficacy

As knowledge around ketamine’s action mechanisms and safety profile solidified, the field saw a pivot towards human studies, particularly double-blind, placebo-controlled trials, to assess its antidepressant efficacy. The exploration extended into pharmacological principles and clinical applications, employing meta-analysis to validate ketamine’s effectiveness for specific conditions, indicating a move from purely biological inquiries to clinical relevance.

#### 4.2.4 Emerging focus areas

Research interest has gradually expanded to include the application of ketamine in pediatric populations and its intravenous administration, scrutinizing dose-dependent efficacy changes. Besides, comparative studies evaluating ketamine against other anesthetics like dexmedetomidine, reflecting a growing interest in ketamine’s utility across different medical scenarios. Then, a pronounced focus on ketamine’s antidepressant capabilities, shifting from general anesthetic uses to its potential in treating depressive symptoms, suicidal ideation, anxiety, major depressive disorder, and treatment-resistant depression. Meanwhile, the structural-functional relationship of ketamine, leading to the development of esketamine through enantiomer research, aimed at harnessing ketamine’s therapeutic benefits while minimizing its adverse effects.

Ketamine occupies a pivotal position in various medical domains, notably in anesthesia, where it is distinguished by a broad spectrum of therapeutic effects. It possesses hypnotic, amnesic, analgesic, anti-inflammatory, and sympathomimetic attributes, facilitating dissociative anesthesia. This unique pharmacological profile enables the preservation of laryngeal reflexes alongside the maintenance of respiratory and cardiovascular stability (Ketamine a familiar drug we trust, 2011). Most importantly, ketamine does not result in respiratory depression, and patients maintain hypercapnic reflexes and residual functional capacity with moderate bronchodilator effects (von Ungern-Sternberg et al., 2007). In the treatment of psychiatric disorders, as an antidepressant treatment, ketamine has gained attention for its rapid onset of action and for its ability to rapidly reduce depressive symptoms, especially in patients with refractory depression (Andrade, 2017). Ketamine’s therapeutic efficacy extends beyond major depressive disorder (MDD) to encompass the treatment of obsessive-compulsive disorder (OCD), social anxiety disorder, and post-traumatic stress disorder (PTSD), conditions frequently comorbid with MDD and potentially exacerbating treatment resistance. A seminal study by Berman et al. in 2000 highlighted ketamine’s remarkable capacity for rapid symptom alleviation in psychiatric care. The administration of a single intravenous dose (0.5 mg/kg) of racemic ketamine to patients diagnosed with MDD resulted not only in an immediate antidepressant effect but also sustained this effect for a minimum of 72 h (Berman et al., 2000). Subsequent research has robustly confirmed the antidepressant and anti-suicidal benefits of single or multiple intravenous administrations of racemic ketamine (0.5 mg/kg) in individuals with treatment-resistant major depressive disorder (MDD) or bipolar disorder (BD) (Wilkinson et al., 2018; Zheng et al., 2018). Despite the promising findings regarding ketamine’s efficacy in treating mood disorders, it is important to note that these results are still preliminary. The majority of the relevant studies conducted thus far are small in scale, indicating a need for further replication to validate these outcomes. Additionally, a recent meta-analysis revealed that ketamine administration did not result in a significant improvement in symptoms of PTSD (Borgogna et al., 2024). Moreover, ketamine is recognized for its neuroprotective properties. A 2018 study illustrated that ketamine administration in cases of acute brain injury diminished neuronal cell death and enhanced cognitive functionality. Furthermore, ketamine has been shown to foster neuroplasticity, thereby augmenting learning and memory capacities. The compound’s potential therapeutic applications extend into the domain of substance use disorders; a comprehensive literature review in 2020 highlighted ketamine’s efficacy in mitigating symptoms associated with various addictions, including alcohol, opioid, and even ketamine dependence itself (Witkin et al., 2020). Furthermore, a review in 2024 has underscored that ketamine can enhance learning capabilities by modulating synaptic connections between neurons (Simonini et al., 2022). However, despite its many benefits, ketamine has adverse side effects associated with psychotomimetic effects (i.e., delirium, confusion, disturbances in perception and thinking, and hallucinations) and sympathomimetic effects (i.e., arterial and intraocular hypertension, and airway mucus hypersecretion) (von Ungern-Sternberg et al., 2007; Ketamine, 2011).

S-ketamine, the S-enantiomer of ketamine, specifically targets and mitigates the neurotransmitter glutamate’s activity in the brain by inhibiting N-methyl-D-aspartate (NMDA) receptors. Exhibiting a three to fourfold higher affinity for these receptors compared to ketamine, S-ketamine boasts more potent analgesic capabilities and reduced psychotomimetic effects (Zanos et al., 2018), rendering it a more selective and biologically efficacious agent. Its utility in the treatment of depression and various psychiatric disorders is underscored by its rapid antidepressant properties. Upon intravenous administration of a 0.5 mg/kg dose, S-ketamine achieves peak plasma concentrations within approximately 67 s and maintains a half-life of roughly 287.50 ± 110.20 min, further evidencing its swift pharmacokinetic profile (Wang et al., 2019). It can produce analgesia at low doses and anesthesia and strong analgesia at high doses (Peltoniemi et al., 2016). Intranasal formulations of the esketamine have demonstrated moderate efficacy in the context of treatment-resistant depression (TRD), typically characterized by inadequate response to at least two distinct trials of antidepressants. In patients with refractory depression and symptoms of major depression, such as suicidal ideation, who are at imminent risk of suicide, a rapid onset of antidepressant effect has been observed in patients with refractory depression at 2 h (Singh et al., 2016) and 24 h after administration of a single dose of intranasal ketamine (Daly et al., 2018) and in placebo in controlled trials, patients with suicidal ideation were significantly more likely to respond to a single dose of intranasal ketamine than to a single dose of placebo at 4 h and 24 h after the first dose. In placebo-controlled trials, patients harboring suicidal thoughts showed a significantly greater likelihood of responding to intranasal ketamine compared to placebo at both 4 and 24 h subsequent to administration (Canuso et al., 2019).

### 4.3 Strengths and limitations

Bibliometric analysis offers a nuanced understanding of ketamine’s historical trajectory and pinpointing nascent research areas, outperforming traditional review methodologies by aggregating and analyzing extant evidence. Unlike conventional reviews, bibliometric analysis leverages publications and their citation data to uncover trends and patterns. However, reliance on these data sources can introduce certain biases, such as citation bias. Factors including self-citation, the perceived authority of authors, and the impact factor of journals may skew these analyses. Consequently, our study emphasizes significant nodes, like highly cited works and pivotal keywords, which tend to be more robust indicators of a field’s core areas and trends, albeit not directly reflective of the quality or relevance of the cited works.

A notable limitation of our approach is the exclusive use of the WOSCC for data gathering, potentially overlooking relevant publications housed in other databases. Additionally, the present state of bibliometric software restricts full-text and citation scrutiny to a few databases, excluding major repositories like Embase and the Cochrane Systematic Review database. This limitation curtails our ability to perform a more integrative analysis across diverse data sources. Despite these challenges, the WOSCC’s extensive literature coverage justifies its selection for bibliometric study. Looking forward, advancements in bibliometric software should aim at enhancing functionality to amalgamate data from varied databases, thus broadening the scope and depth of evidence synthesis and bolstering the utility of bibliometric analyses in guiding future clinical and research endeavors.

## 5 Conclusion

This study marks the inaugural comprehensive review and analytical exploration of the worldwide landscape of ketamine research. Over the past decade, there has been a notable surge in the publication of high-caliber research within this field, with the crescendo of activity reaching its zenith in 2022. Despite these advancements, the field of ketamine research stands on the cusp of further breakthroughs. For continued progress, it is imperative for future investigations to foster international collaborations that transcend geographical boundaries, integrating seminal findings to deepen our understanding of ketamine’s clinical applications. Such collaborative endeavors should focus on refining ketamine’s indications and dosages across diverse patient demographics to maximize therapeutic efficacy while minimizing risk. Furthermore, esketamine, a stereoisomer of ketamine, presents a promising avenue for research, offering the possibility of reducing adverse effects inherent to ketamine while preserving its therapeutic benefits. Investigating esketamine’s utility in various clinical contexts represents a critical next step in the evolution of ketamine research, promising to expand the therapeutic arsenal available for managing complex medical conditions. This study not only charts the trajectory of ketamine research to date but also sets the stage for future inquiries aimed at unlocking the full therapeutic potential of ketamine and its derivatives.

## Author contributions

YW: Writing – original draft, Data curation, Formal analysis, Investigation, Methodology, Software, Visualization. SC: Writing – original draft, Formal analysis, Investigation, Methodology, Visualization. DC: Writing – review & editing, Conceptualization, Investigation, Supervision, Validation.
